# Estrogen receptor 1 gene (TA)n polymorphism is associated with lone atrial fibrillation in men

**DOI:** 10.3325/cmj.2014.55.38

**Published:** 2014-02

**Authors:** Karlo Golubić, Anton Šmalcelj, Jadranka Sertić, Ljiljana Juričić

**Affiliations:** 1 Department of Cardiovascular Diseases, University Hospital Center, University of Zagreb, School of Medicine, Zagreb, Croatia; 2 Department of Cardiovascular Diseases, University Hospital Center, University of Zagreb, School of Medicine, Zagreb, Croatia; 3 Laboratory for Molecular Diagnostics, Department of Laboratory Diagnostics, University Hospital Center, University of Zagreb, Zagreb, Croatia; 4 Laboratory for Molecular Diagnostics, Department of Laboratory Diagnostics, University Hospital Center, University of Zagreb, Zagreb, Croatia

## Abstract

**Aim:**

To determine the association between the number of thymine-adenine (TA)n dinucleotide repeats in the promoter region of the gene coding for the estrogen receptor alpha (ESR1) and the prevalence of lone atrial fibrillation (AF) in men.

**Methods:**

We conducted a case-control study involving 89 men with lone AF and 166 healthy male controls. The ESR1 genotype was established by polymerase chain reaction and capillary electrophoresis. To assess the association of ESR1 genotype with AF, logistic regression models were built with AF as outcome.

**Results:**

Men with lone AF had significantly greater number of (TA)n repeats of single alleles than controls (mean ± standard deviation, 19.2 ± 4.2 vs 18 ± 4.3, *P* = 0.010). After adjustment for other factors, a unit-increase in (TA)n repeat number was associated with a significantly greater likelihood of AF (odds ratio 1.069; 95% confidence interval 1.024-1.116, *P* = 0.002).

**Conclusions:**

Our results indicate that a greater number of (TA)n repeats in the promoter region of ESR1 is associated with a significantly increased likelihood of lone atrial fibrillation in men.

Atrial fibrillation (AF) is considered the most common sustained arrhythmia of clinical importance (1). Even if corrected for etiological factors such as ischemic and hypertensive heart disease, its prevalence in men is 1.5 times higher than in women (1). lone AF is defined as a normal echocardiogram and no clinical history of known etiological factors for AF and it occurs in the normal heart without any known causal factor (2). It has similar sex prevalence as AF, and such prevalence may indicate the effects of sexual hormones on cardiac electrophysiology through the corresponding intracellular steroid receptors (3).

Genetic determinants of AF have been explored using different methods, ranging from candidate gene studies investigating rare mutations (4) with alterations in cardiomyocite structure and function (mostly ion channel alterations) to genome-wide association studies examining subtle effects of single nucleotide polymorphisms (5). Nucleotide polymorphisms have been widely investigated, mostly those affecting ion channel function, cardiac renin-angiotensin-aldosterone system, inflammatory responses, and cardiac connexins (6-12)

Less attention, however, has been paid to the association of AF and nucleotide polymorphisms of cardiac sexual steroid receptors. Sexual steroid hormones exert complex genomic and non-genomic cardiac effects through corresponding receptors (13). The gene coding for the estrogen receptor α (ESR1) is located on chromosome 6 (6q25–27). It comprises eight exons and has a polymorphic thymine-adenine (TA) region located 1174 base pairs (bp) upstream from the exon 1 in the genes promoter region. Out of many ESR1 polymorphisms, alleles with a larger number of (TA)n repeats have received the most research attention, making it one of the most promising research targets (14). A previous study suggested a positive correlation between homozygosity for longer alleles (≥19 TA repeats) and AF (15). Therefore, we aimed to examine the association between the actual number of (TA)n repeats and lone AF in men using a case-control design.

## Materials and methods

### Study population

**Figure 1:**
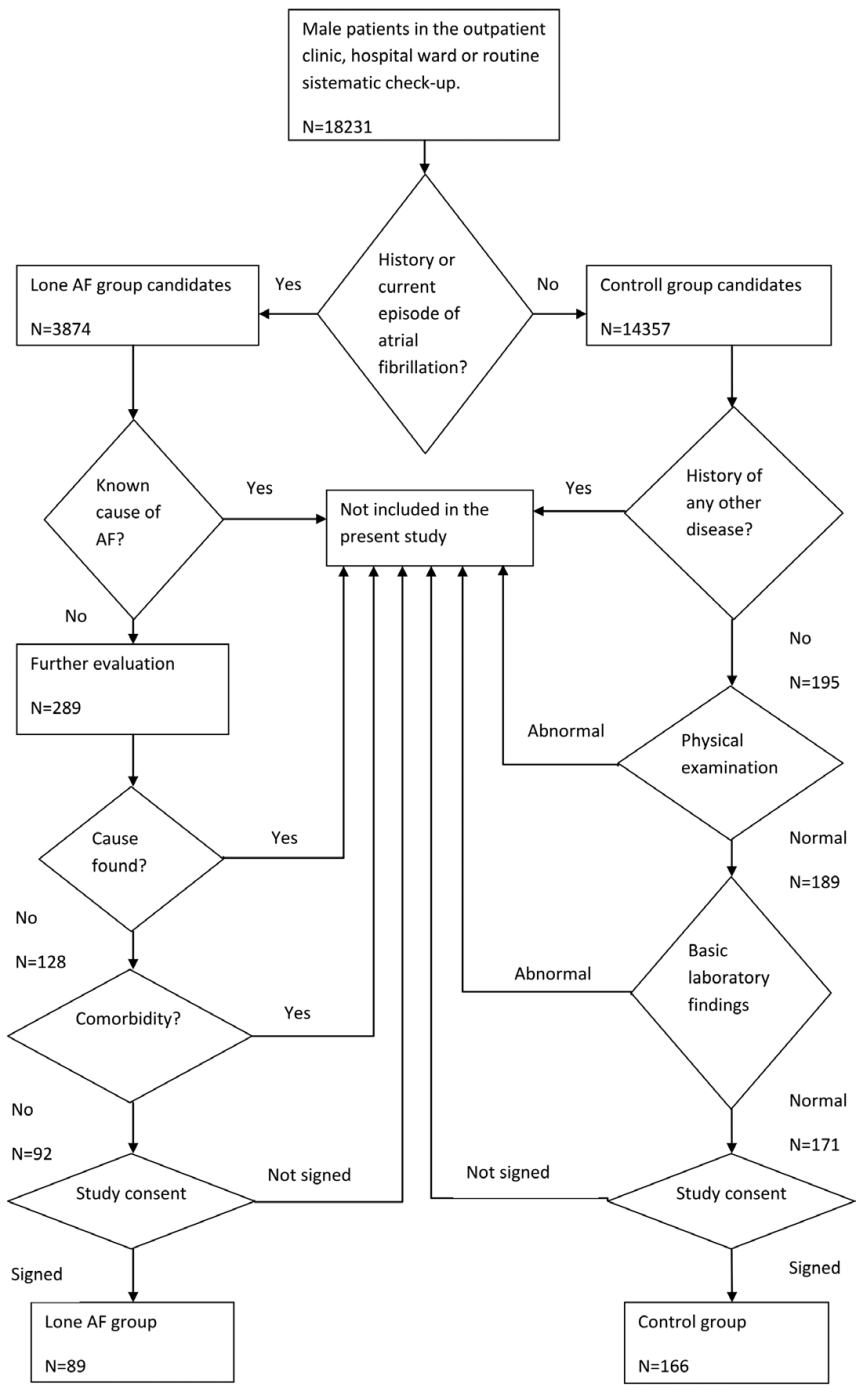
The stepwise exclusion process of cases and controls.

This case-control study included 89 male patients with lone AF and otherwise structurally and functionally normal hearts and 166 healthy male participants without any known disease. All participants were of Caucasian origin recruited from the city of Zagreb and the wider region and were included in the study as they appeared in our hospital or outpatient clinic from January 2010 to December 2012 (Figure 1). Informed consent was obtained from each person and the study protocol conformed to the ethical guidelines of the 1975 Declaration of Helsinki. The study was approved by the Medical Ethics Committee of the hospital.

### Cases

The main inclusion criterion for the AF group was electrocardiographically (ECG) documented AF in the absence of known etiological factors for AF (ie, hypertension, symptomatic heart failure, valvular heart diseases, cardiomyopathies, congenital heart defects, coronary artery disease, thyroid dysfunction, obesity, diabetes mellitus, chronic obstructive pulmonary disease, sleep apnea, and chronic renal disease) (16). All AF patients underwent a non-invasive cardiologic evaluation, including echocardiography. Patients with a history of smoking, drug use, and alcohol intake were excluded (Figure 1).

### Controls

The control group consisted of volunteers from the preventive health check program with healthy hearts and normal electrocardiograms. On physical examination they were found to have no signs or symptoms of any disease, as people with not normal test results were excluded from the study (Figure 1) (Supplementary Table)(supplementary table). The necessary sample size was calculated beforehand using expected frequencies from our previous research, 2:1 control to case ratio, type I error probability of 0.05, and statistical power of 80% by standard methods assuming that outcome data will be analyzed prospectively by uncorrected χ2 test. The calculations yielded 65 cases and 130 controls. Sample size calculation for logistic regression was obtained by the Peduzzi method for 4 covariates and prevalence of 35%. The minimum required total sample size (cases + controls) was 114. Our sample of 255 was thus large enough for adequate analysis.

### Methods

Height was measured using a Harpender stadiometer and weight on a digital scale without shoes. Body mass index (BMI) was calculated as the weight in kilograms divided by the square of height in meters. Blood pressure was measured in a sitting position using a standard analog sphygmomanometer. Mean arterial pressure (MAP) was calculated by the formula: systolic blood pressure (SBP) × 1/3 + diastolic blood pressure (DBP) × 2/3. The serum estradiol level was assessed by a competitive immunoassay technique (Ortho Johnson & Johnson, High Wycombe, UK).

### Echocardiographic data

The transthoracic echocardiogram was performed using a GE Vivid 7 echocardiograph (GE Healthcare, Little Chalfont, UK). All examinations were performed by cardiologists with accreditation in transthoracic echo-cardiography by the European Society of Cardiology. The images were later reanalyzed using the GE Health Care EchoPac Dimension software, PC version 108.1.3 (GE Healthcare, Little Chalfont, UK).

### Assessment of ESR1 genotype

Genomic DNA was extracted from peripheral blood leucocytes using the Nucleon II DNA extraction kit (Scotlab, Coatbridge, Lanarkshire, UK) according to the manufacturer’s instructions. Analysis was performed within one week after the blood sample was obtained.

Dinucleotide polymorphism of ESR1 was analyzed using polymerase chain reaction (PCR) amplification with labeled primer 5′-6-FAM-GAC GCA TGA TAT ACT TCA CC-3′ and 5′ - GCA GAA TCA AAT ATC CAG ATG-3′. PCR was performed with 25 cycles consisting of 2 minutes at 94°C, 1 minute at 58°C, and 1 minute at 72°C, followed by 30 minutes at 60°C after the last cycle. The alleles were size-separated by capillary gel electrophoresis using Gene Scan Fragment Analysis Software 4.0 (Applied Biosystems, Foster City, USA). In short, 1 μL of the product was diluted with 12 μL of deionized formamide containing 0.5 μL GeneScan-500 ROX internal lane standard for sizing DNA fragments. Capillary electrophoresis was then carried out using ABI PRISM 310 Genetic Analyzer and POP-6 (Applied Biosystems, Foster City, CA, USA), which had been created for applications requiring high resolution under denaturing electrophoretic conditions (17).

### Statistical analysis

Data are shown as mean values with the standard deviation (SD) for continuous variables and absolute numbers with prevalence (%) for categorical variables. Departure from normal distribution was initially evaluated for all continuous variables by Kolmogorov-Smirnov test and graphically. Mann-Whitney U test and Kruskal-Wallis test were conducted to compare the differences between cases and controls for continuous variables and χ^2^ test for categorical variables. To assess the association of ESR genotype with AF, binary logistic regression models were built with AF as outcome. Model 1 was unadjusted, whereas Model 2 was adjusted for age, MAP, and serum estradiol concentration. Likelihood ratio test was used to determine the significance of odds ratios. For all analyses, we used statistical package STATISTICA, version 9.1. (*www.statsoft.com*) and a double-sided *P* < 0.05 was considered significant.

## Results

**Figure 2:**
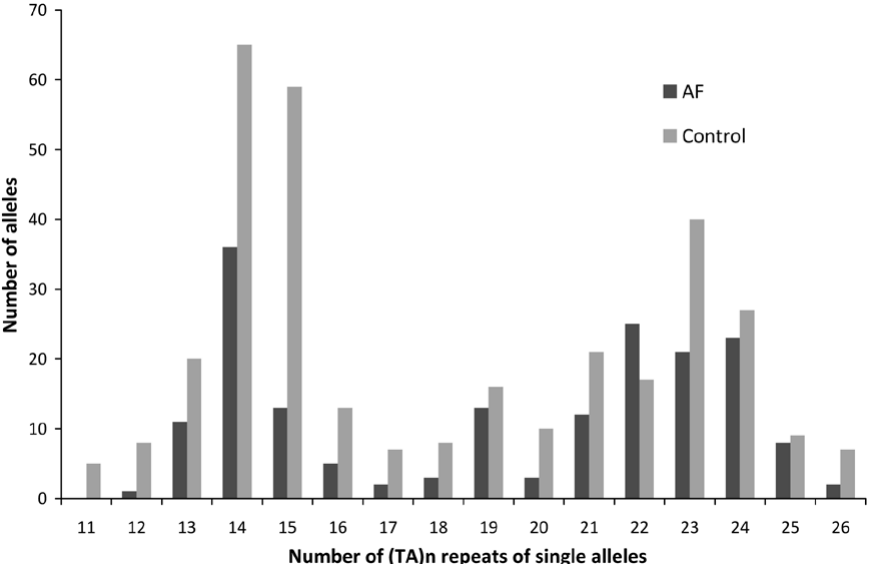
Bimodal distribution of (TA)n repeats in cases and controls.

There was no significant difference in clinical, echocardiographycal, and biochemical characteristics between cases and control group (Table 1). We observed 16 different alleles with (TA)n repeat number ranging between 11 and 26. This polymorphism showed a bimodal distribution, with two peaks – at 14 repeats (19.2% of alleles) and 23 repeats (12% of alleles), and a breakpoint at 17 and 18 repeats as described in earlier studies (18). The same bimodal pattern emerged when we analyzed the distribution of TA repeats by group (Figure 2).

**Table 1:** Clinical, echocardiographycal, and hormonal characteristics of cases and controls

	Atrial fibrillation cases (N = 89)	Controls (N = 166)	*P* value*
Age (years)	47.93 ± 11.76	46.34 ± 10.02	0.124
Systolic blood pressure (mmHg)	119.8 ± 7.3	119.4 ± 7.2	0.660
Diastolic blood pressure (mmHg)	77.3 ± 4.8	77.5 ± 4.7	0.803
Body mass index (kg/m^2^)	23.1 ± 3.1	22.9 ± 2.9	0.629
End-diastolic diameter of the left ventricle (cm)	5.09 ± 0.34	5.05 ± 0.33	0.865
Interventricular septum thickness (cm)	0.94 ± 0.15	0.92 ± 0.16	0.768
Posterior wall (of the left ventricle) thickness (cm)	0.84 ± 0.15	0.85 ± 0.14	0.840
Ejection fraction (of the left ventricle) (%)	66 ± 5.0	66 ± 4.5	0.775
Left atrium transverse axis length (end-systolic) (cm)	3.85 ± 0.66	3.95 ± 0.52	0.757
Left atrium volume (end- systolic) (mL)	41.74 ± 20.1	41.64 ± 19.2	0.816
Right atrium volume (end-systolic) (mL)	30.42 ± 14.71	30.37 ± 13.3	0.854
Estradiol (pmol/L)	54.8 ± 12.2	55.2 ± 11.3	0.726

*Continuous variables were compared using two-tailed Mann-Whitney test.

The ESR1 gene promoter TA repeats were classified as short (≤18 TA repeats, 160-176 bp) and long (≥19 TA repeats, 178-194 bp), corresponding to their bimodal distribution and in accordance with previous studies on cardiovascular diseases and non-cardiovascular diseases (18). Consequently, 3 possible genotypes were studied: SS (both alleles short), LL (both alleles long), and SL (mixed, one long and one short allele). Men with lone AF had a higher prevalence of homozygous LL combination than the control group (35 [39.33%] vs 33 [19.88%], *P* = 0.003, χ^2^ = 11.7, χ^2^ test), while the prevalence of the SL combination (36 [40.45%] vs 81 [48.8%], *P* = 0.202, χ^2^ = 1.625, χ^2^ test) and the SS combination (18 [20.22%] vs 52 [31.33%], *P* = 0.058, χ^2^ = 3.585, χ^2^ test) were lower in the AF group.

**Figure 3:**
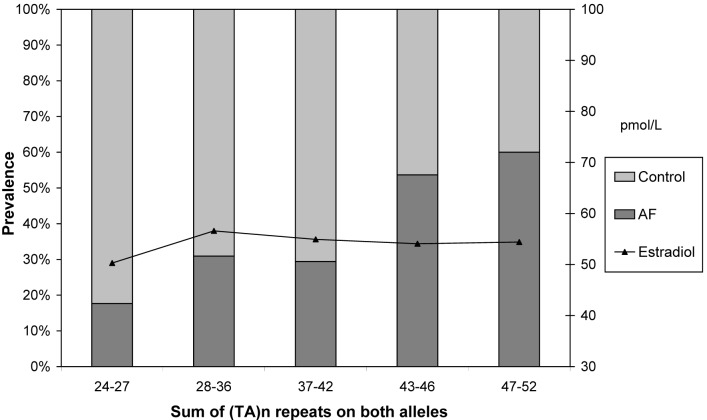
The association between the prevalence of lone atrial fibrillation and sums of (TA)n repeats of both alleles.

In order to clarify the relationship between the allele length and phenotype, we compared the number of (TA)n repeats in single alleles between the AF and control group and found a significant difference (19.2 ± 4.2 vs 18 ± 4.3, Mann-Whitney U test, two-tailed, *P* = 0.010). We also created (post hoc for analysis purposes) five groups of examinees based on the sum of (TA)n repeats of both of their alleles to be used as predictors and observed greater prevalence of AF with a greater sum of (TA)n repeats in a dose-dependent manner (χ^2^ = 14.537, *P* = 0.006), while serum estradiol concentration did not differ significantly between the groups (Kruskal-Wallis test: H = 4.93, df = 4, *P* = 0.295) (Figure 3).

To test whether the observed relationship was confounded by already known risk factors of AF, logistic regression was performed (Table 2). In Model 1 (unadjusted), there was a 6.9% significantly greater likelihood of AF associated with a unit-increase in (TA)n repeat number of single alleles, OR = 1.069 (95% CI, 1.024-1.116, *P* = 0.002). When adjustment was made for age, blood pressure and serum estradiol concentration were added (Model 2), the observed association remained significant and its magnitude did not change.

**Table 2:** Association between (TA)n repeats and lone atrial fibrillation in men

Models	Variable	Odds ratio	95% confidence interval	*P*
Model 1 (univariable)	number of (TA)n repeats	1.069	1.024-1.116	0.002
Model 2 (multivarable)	number of (TA)n repeats	1.072	1.026-1.119	0.002
	mean arterial pressure (mmHg)	1.006	0.958-1.056	0.809
	age (years)	1.016	0.998-1.034	0.073
	serum estradiol concentration (pmol/L)	0.997	0.981-1.013	0.692

## Discussion

Our results suggest a positive association between the number of (TA)n repeats in the promoter region of ESR1 and the prevalence of lone AF in men. The significance of the observed relationship after adjustment for age, serum estradiol, and blood pressure indicates that the effect of (TA)n is independent of these putative confounders.

The basic role of estrogens in both sexes is modulation of immune responses, oxidative stress, inflammation, cellular growth, proliferation and apoptosis, and affecting the central nervous, cardiovascular, and skeletal systems (13,19). Evidence suggests extragonadal production of sexual steroid hormones in the heart and blood vessels, as well as local effects of estrogens in the male heart, which are converted from testosterone by cytochrome P450 aromatase (20).

The proposed mechanisms by which sex steroids affect cardiac electrophysiology include their effect on nuclear and extranuclear receptors in cardiomyocites (3,21,22), modulating gene expression via many signaling pathways (23), but also non-genomic actions like influencing ion channel conductance and altering cell potentials (24).

ESR1 repeats are only one of many ESR1 polymorphisms (14) and their phenotypic expression is subtle (25). The molecular mechanism by which (TA)n repeat polymorphism may be associated with AF is unclear. The ESR1 gene has a very complex genomic organization containing multiple promoter regions with alternative splice sites, resulting in the expression of alternative first exons and different ER-α transcripts (26). It has been suggested that (TA)n dinucleotide repeat length may affect alternative promoter usage, resulting in unsuitable ER-α expression in certain tissues (27). In addition, a regulatory enhancer element that may act as a steroid response element has been identified approximately 200 base pairs downstream of the (TA)n repeat (28). Although the role of this enhancer is not yet clear, its proximity to the polymorphic repeat region makes it a potential target for functional affects of (TA)n repeat number. It is also possible that the number of (TA)n repeats does not influence the heart directly, but via linkage disequilibrium with other coding regions.

Electrophysiological research on ESR1 polymorphisms has so far been mostly related to ventricular rather than to atrial arrhythmias. To our knowledge, this is the second report of the effect of the (TA)n repeat ESR1 polymorphism on atrial fibrillation, the first one being a small pilot-study that suggested a correlation between homozygosity for alleles with a larger number of (TA)n repeats and AF (15). Studies in other fields have shown that more (TA)n repeats lead to a less chemically active estrogen receptor, which is in agreement with our study (17,29).

Our study has several limitations. We were able to analyze only the (TA)n repeat polymorphism. It is possible that other polymorphisms of the same gene or polymorphisms of the gene encoding the estrogen receptor β could also be associated with lone AF. The study was limited to male participants so the results may not be applicable to women.

Our results indicate that a greater number of (TA)n repeats in the promoter region of ESR1 is associated with an incremental increase in the likelihood of lone AF in men. This result could give new insights into the role of steroid hormones in the pathogenesis of AF. Large-scale studies conducted in different populations are required to more comprehensively and reliably assess the relationship of (TA)n length with the quantity and quality of ER-α transcripts, and the occurrence of AF.
