# Supplementary material for: Estrogen receptor 1 gene (TA)n polymorphism is associated with lone atrial fibrillation in men
Source: Croat Med J. 2014 Feb;55(1):38–44. doi: 10.3325/cmj.2014.55.38 (PMC3944416; doi:10.3325/cmj.2014.55.38)
Supplement: Supplementary Table 1 [file CroatMedJ_55_s003.pdf]

Table 1. Normal basic laboratory values used for exclusion of unhealthy individuals

Variable	Normal range
Eritrocyte count	4.34-5.72 $\times 10^{12}$/L
Leukocyte count	3.4-9.7 $\times 10^{9}$/L
Neutrophil granulocytes	2.06-6.49 $\times 10^{9}$/L
Lymphocytes	1.19-3.35 $\times 10^{9}$/L
Monocytes	0.12-0.84 $\times 10^{9}$/L
Eosinophil granulocytes	0.00-0.43 $\times 10^{9}$/L
Basophil granulocytes	0.00-0.06 $\times 10^{9}$/L
Thrombocyte count	158-424 $\times 10^{9}$/L
Hemoglobin	138-175 g/L
Hematocrit	0.415-0.530
Mean corpuscular volume	83.0-97.2 fL
Mean corpuscular hemoglobin	27.4-33.9 pg/cell
Mean corpuscular hemoglobin concentration	320-345 g/L
Red blood cell distribution width	9-15 %
C-reactive protein	<5 mg/L
Sodium	137-146 mmol/L
Potassium	3.9-5.1 mmol/L
Urea	2.8-8.3 mmol/L
Creatinine	79-125 μmol/L
Fasting blood glucose	4.4-6.4 mmol/L
Bilirubin - total	3-20 μmol/L
Alkaline phosphatase	60-142 U/L
Alanine transaminase	12-48 U/L

Aspartate transaminase	11-38 U/L
Gamma-glutamyltransferase	11-55 U/L
Creatine kinase	0-177 U/L
Lactate dehydrogenase	<240 U/L
